# Intravenously Administered Alphavirus Vector VA7 Eradicates Orthotopic Human Glioma Xenografts in Nude Mice

**DOI:** 10.1371/journal.pone.0008603

**Published:** 2010-01-06

**Authors:** Jari E. Heikkilä, Markus J. V. Vähä-Koskela, Janne J. Ruotsalainen, Miika W. Martikainen, Marianne M. Stanford, J. Andrea McCart, John C. Bell, Ari E. Hinkkanen

**Affiliations:** 1 Department of Biochemistry and Pharmacy, Åbo Akademi University, Turku, Finland; 2 Ottawa Health Research Institute, Ottawa, Canada; 3 A. I. Virtanen Institute, Department of Biotechnology and Molecular Medicine, University of Kuopio, Kuopio, Finland; 4 Division of Experimental Therapeutics, Toronto General Research Institute, Toronto, Canada; Karolinska Institutet, Sweden

## Abstract

**Background:**

VA7 is a neurotropic alphavirus vector based on an attenuated strain of Semliki Forest virus. We have previously shown that VA7 exhibits oncolytic activity against human melanoma xenografts in immunodeficient mice. The purpose of this study was to determine if intravenously administered VA7 would be effective against human glioma.

**Methodology/Principal Findings:**

*In vitro*, U87, U251, and A172 human glioma cells were infected and killed by VA7-EGFP. *In vivo*, antiglioma activity of VA7 was tested in Balb/c nude mice using U87 cells stably expressing firefly luciferase in subcutaneous and orthotopic tumor models. Intravenously administered VA7-EGFP completely eradicated 100% of small and 50% of large subcutaneous U87Fluc tumors. A single intravenous injection of either VA7-EGFP or VA7 expressing *Renilla* luciferase (VA7-Rluc) into mice bearing orthotopic U87Fluc tumors caused a complete quenching of intracranial firefly bioluminescence and long-term survival in total 16 of 17 animals. In tumor-bearing mice injected with VA7-Rluc, transient intracranial and peripheral *Renilla* bioluminescence was observed. Virus was well tolerated and no damage to heart, liver, spleen, or brain was observed upon pathological assessment at three and ninety days post injection, despite detectable virus titers in these organs during the earlier time point.

**Conclusion:**

VA7 vector is apathogenic and can enter and destroy brain tumors in nude mice when administered systemically. This study warrants further elucidation of the mechanism of tumor destruction and attenuation of the VA7 virus.

## Introduction

During the past decades malignant gliomas have presented with an insurmountable obstacle in cancer treatment. While recent results of a multi-center phase III clinical trial combining radiotherapy (RT) with the DNA-alkylating agent temozolomide (TMZ) have marked a true paradigm shift in treatment of glioblastoma multiforme (GBM) [1], the most common type of malignant glioma, patients rarely live beyond two years after diagnosis and there are no cures. Radical surgery can alleviate the effects of excess tumor burden and prolong meaningful life-span, but remains palliative only [2].

One of the biggest reasons for the grim prognosis is the highly invasive nature of gliomas. Glioma cells can migrate long distances in the brain parenchyma and establish satellite lesions even in the contralateral hemisphere [3]. Other reasons for treatment failure are the vast genotypic and phenotypic heterogeneity of malignant gliomas, limiting the effectiveness of any single therapeutic on a population-wide scale, and the presence of tumorigenic cells capable of both resisting conventional therapies as well as escaping immune surveillance [4]. Recently, tumor-initiating cells (TICs) were indentified in malignant gliomas [5]. These cells were later shown to be resistant to ionizing radiation which could well explain why radiation therapy fails to kill malignant gliomas [6].

Oncolytic viruses hold great promise as glioma-killing agents. Reovirus [7], Newcastle disease virus (NDV) [8], Herpes simplex virus-1 (HSV-1) [9], [10], adenovirus [11], vesicular stomatitis virus (VSV) [12], measles virus [13], myxoma virus [14], poliovirus [15] and vaccinia virus [16] have emerged as potential vehicles to combat malignant glioma and have shown oncolytic activity against human glioma xenografts. However, while oncolytic viruses must be able to kill cancer cells, they should leave normal healthy cells unharmed. To achieve this, HSV-1 [10], adenovirus [17] and vaccinia virus [18] have been engineered to selectively replicate only in tumor cells. Other oncolytic viruses including reovirus, measles virus, VSV and NDV, seem to have an inherent capability to specifically replicate only in tumor cells, leaving normal cells largely unaffected. The tumor permissiveness of viral replication is often due to defects in the immunological/antiviral status of the tumor cells. Defects in the interferon response of tumor cells seem to explain the tumor-specificity of VSV [19] and NDV [20]. Reovirus replication, on the other hand, is restricted to cells with activated Ras signaling pathway [21].

Semliki Forest virus (SFV) is an enveloped, positive stranded RNA virus of the family *Togaviridae* [22]. The natural hosts of SFV are small rodents and birds while mosquitoes are the usual vector. Human infections are also common and in certain parts of Africa up to 40% of the population has been shown to be seropositive [23]–[25]. Semliki Forest virus infection does not associate with any disease in humans but it may cause mild symptoms such as headache, fever and rash [23]. Several different strains of SFV exist, some of which are highly virulent for laboratory mice, while others are virulent only for neonatal mice. The A7(74) strain of SFV is a naturally attenuated strain [26]–[28] which, like the virulent wild type strains L10 and SFV4, is neurotropic, infecting both neurons and glial cells, but unlike the these strains which induce fatal encephalitis in mice irrespective of their age, A7(74) is lethal only for newborn mice [26], [29]. In mice older than two weeks, replication of A7(74) in neurons is severely restricted and the mice survive the infection without neurological sequealae [29]. The mechanism of neuronal restriction of replication is yet unknown but has been shown to be independent of type I interferon signaling [30].

We have constructed a replication-competent expression vector VA7-EGFP based on the A7(74) virus [31]. VA7-EGFP has been shown to replicate in and kill a number of cultured tumor cells similar to SFV4 and to be able to deliver therapeutic genes into the CNS of diseased mice upon intraperitoneal injection [31]. In a recent report, we have described the oncolytic potential of VA7-EGFP against human melanoma xenografts in severe combined immunodeficient (SCID) mice [32].

Given the oncolytic potential and CNS tropism of VA7 upon peripheral administration, it was of interest to investigate whether VA7 would be able to inhibit the growth of human glioma xenografts implanted either subcutaneously or orthotopically in nude mice. In the present report we show that intravenously administered VA7 virus effectively eradicated both subcutaneous and orthotopic U87MG tumor xenografts in nude Balb/c mice. Besides initial transient viremia none of the mice displayed any neurological symptoms or signs of pathology.

## Results

### VA7-EGFP–Induced Oncolysis in Glioma Cell Lines

**Figure 1:**
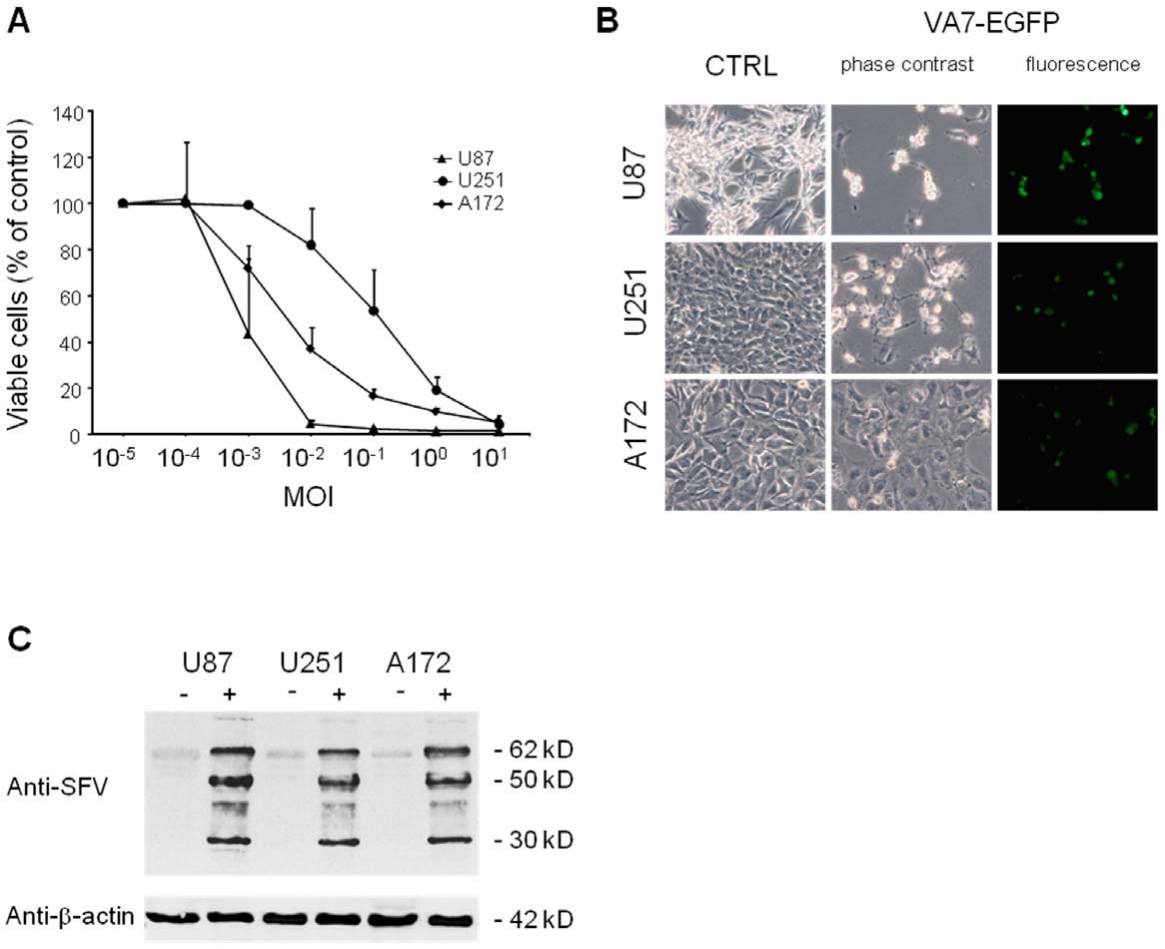
VA7-EGFP kills glioma cells *in vitro*. (A) Dose-dependent killing by VA7-EGFP of U87, U251 and A172 glioma cells in culture. Glioma cells were seeded in a 96-well plate and VA7-EGFP at MOI ranging from 10^−5^ to 10^1^ was added 16 hours later. Cell viability was assessed 96 hours later using calcein AM as indicator of cell membrane integrity. The mean values and standard deviations are shown. (B) Photograph of the cells at 96 hours post infection with VA7-EGFP at MOI 0.1. Fluorescence channel for VA7-EGFP shows GFP-positive cells indicative of active virus replication. (C) Western blot of VA7 structural protein expression in untreated and VA7-EGFP -infected cells 18 hours post infection. Equal number of cells were infected at MOI 1 and collected for Western analysis 18 hours later. In all cell lines the 30 kDa capsid, the E1 and E2 50 kDa proteins and the E2 precursor p62 were abundantly expressed.

U87, U251 and A172 cell lines were all infected by VA7-EGFP (Figure 1B) but the rate of spread was different in each one. Cell viability assay showed that U87 cells were killed within 96 hours at very low MOI values with 42% of the cells being alive at MOI 0.001 and at MOI 0.1 only 1.8% of the cells were viable at this time point (Figure 1A). U251 cells required a 100-fold higher viral dose to achieve a similar degree of cell death with 53.4% of the cells alive at MOI 0.1 and 4.1% alive at MOI 10. The sensitivity of A172 cells to VA7-EGFP fell between the other two glioma cell lines with 36.4% of the cells being viable at MOI 0.01 (Figure 1A). Viral replication in the infected cells was confirmed by Western blot analysis of viral structural proteins. In all cell lines, the 30 kDa capsid, 50 kDa E1 and E2 proteins as well as the p62 precursor protein were abundantly expressed, confirming productive infection (Figure 1C).

### Characterization of Luciferase Expressing U87 Cells

U87 cells transduced with the lentiviral vector WPTFluc at MOI 1, 5, 25 and 50 exhibited Fluc activity which increased linearly with increasing virus dose (Figure S1A). Fluc expression did not affect cell proliferation as compared to non-transduced control cells (Figure S1B) and we chose to use U87 cells that had been transduced at MOI 25 for subsequent studies. Fluc expression did not change the sensitivity of the U87Fluc cells to VA7-EGFP –induced cell killing as compared to parental U87 cells (Figure S1C). Flow cytometry analysis showed that at least 94% of the cells in U87Fluc cell population were Fluc positive (Figure S1D).

### Oncolytic Efficacy of VA7-EGFP in Subcutaneous Nude Mouse Model

**Figure 2:**
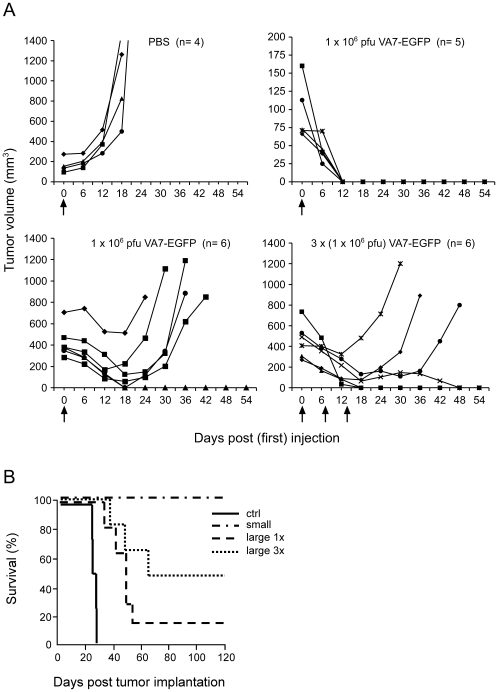
Effect of intravenous injection of VA7-EGFP on the growth of subcutaneous U87Fluc tumor xenografts. (A) U87Fluc cells (5×10^6^) were implanted subcutaneously in nude mice and when the tumors had grown to an average size of 100 mm^3^, the animals were given a single intravenous injection of either PBS or 1×10^6^ PFU of VA7-EGFP. In another two groups, the tumors were allowed to grow until they reached an average size of 450 mm^3^ and the animals were given either a single intravenous injection of 1×10^6^ PFU of VA7-EGFP or three injections of the virus at 7-day intervals, each 1×10^6^ PFU. (B) Kaplan-Meier survival analysis of mice with subcutaneous U87Fluc xenografts and treated with either PBS or intravenous injections of VA7-EGFP.

When the subcutaneous tumors had reached average sizes of either 96.5 mm^3^ (small) or 442 mm^3^ (large), the mice were injected intravenously in the tail vein with 10^6^ PFU of VA7-EGFP. Median survival of mice treated with PBS was 26 days (95% CL = 25 days to 28 days) at which time point the tumor size had reached 1040 mm^3^ (95% CL = 732 mm^3^ to 1344 mm^3^) (Figure 2A, B) and the animals were sacrificed. For the small tumors, a single injection of the virus led to complete and lasting tumor eradication in all of the 5 treated animals over a 4-month follow-up period (Figure 2A). A single intravenous injection of the virus in mice harboring large tumors produced one full cure out of six with a median survival time of 49 days (95% CL = 41 days to 53 days) and a significant increase in survival compared to the PBS-treated group (log-rank test, *P*<0.01) (Figure 2A,B). Three weekly injections with VA7-EGFP yielded three out of six cures (median survival not reached), also with a significant increase in survival (log-rank test, *P*<0.005) (Figure 2A,B). VA7-EGFP did not induce any neurological or other symptoms in the treated mice, and no effect on animal weight was observed (Figure S2).

**Figure 3:**
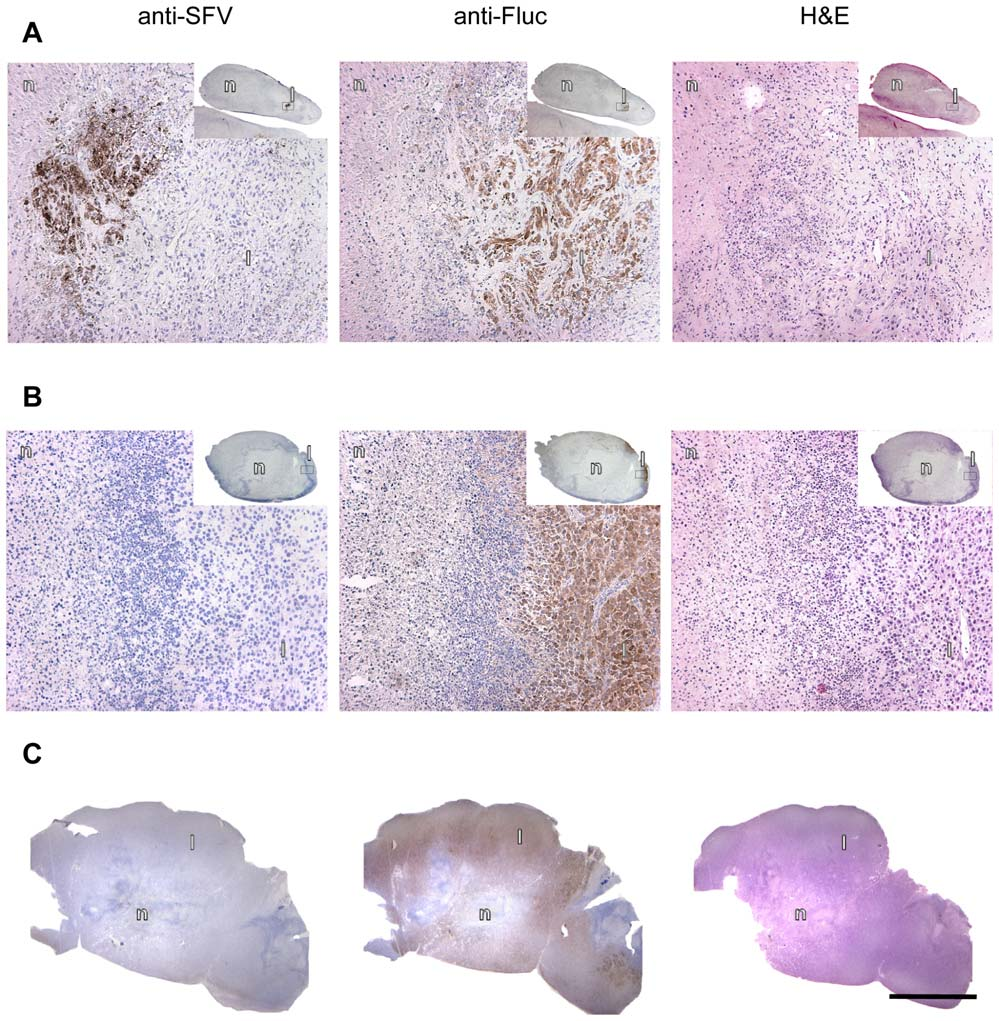
Histological analysis of subcutaneous U87Fluc tumor xenografts. Anti-SFV immunostaining, anti-luciferase staining and H&E staining, respectively, of parallel sections of residual tumor tissue in a mouse 7 days after injection with a single intravenous dose of 1×10^6^ PFU of VA7-EGFP (A) or 21 days after the third injection of VA7-EGFP (B) (original magnification ×200, insert ×10). (C) Similar staining of a xenograft from a PBS-treated tumor-bearing mouse. n = necrotic tissue, l = live cells. Bar = 5 mm.

Histological examination of tumors isolated 7 days after a single injection with VA7-EGFP showed wide-spread necrosis with only very few regions of Fluc positive live tumor cells which were located next to an SFV-positive region implying that the viral infection was still proceeding (Figure 3A). While those xenografts that regrew after three weekly doses of virus were large at study end point, Fluc positive cells were still restricted to the periphery of the tumors (Figure 3B). In parallel HE-stained sections the bulk of the tumors appeared necrotic (Figure 3B, insert). In contrast, tumors isolated from untreated animals showed widespread distribution of live, Fluc positive tumor cells with necrotic areas detected mainly in the very core of the tumor sample (Figure 3C).

### Assessment of the Presence of Virus-Resistant Cells in Regrowing Tumors

In order to study whether tumor regrowth after initial regression was due to emergence of virus-resistant cells, we established several explant cell lines from treated regrowing tumors as well as PBS-treated tumors, and infected them with different concentrations of stock virus. All explant cell lines were Fluc positive as judged from staining with anti-luciferase antibody (Figure S3A). Despite quantitative differences in the sensitivity to virus-induced cell killing between the explanted cells and the parental U87Fluc cells at MOI values between 0.001 and 0.01, all explant cell lines from treated-tumors were more sensitive to VA7-EGFP than cell lines isolated from PBS-treated tumors (Figures S3B). Hence, the failure of VA7-EGFP to eradicate a portion of the larger tumors was not due to virus-resistant glioma cells as observed previously using VA7-EGFP in a human melanoma model [32].

### Oncolytic Efficacy of VA7-EGFP in Orthotopic Nude Mouse Model

**Figure 4:**
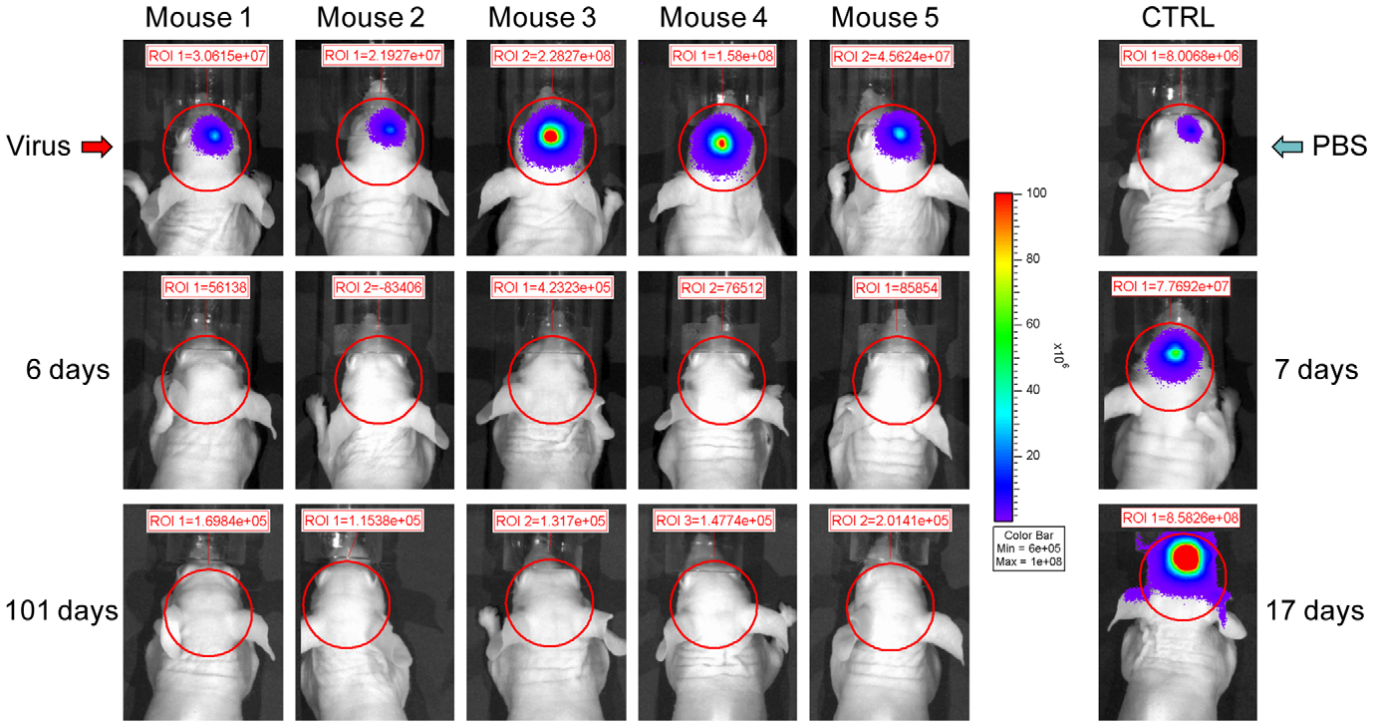
Effect of a single intravenous injection of VA7-EGFP on the growth of orthotopic U87Fluc xenografts. U87Fluc cells (2×10^5^) were implanted stereotactically in nude mice. Tumor growth was monitored by measuring the light produced by the tumor cells with a cooled CCD camera after the animals had been injected with luciferin. Sixteen days post tumor implantation 5 mice harboring intracranial tumors received a single i.v. injection of 1×10^6^ PFU of VA7-EGFP. Tumor signal was extinguished within 6 days and remained below detection limit until the end of the study. A PBS-treated mouse is provided as comparison (CTRL).

Mice with established intracranial tumors assessed by IVIS were either given an intravenous injection of PBS (CTRL) or treated with a single intravenous injection of 1×10^6^ PFU of VA7-EGFP. Median survival of animals treated with PBS was 36 days (95% CL = 26 days to 42 days) at which time the Fluc end point value of 10^9^ photons/sec/cm^2^/steradian was reached (approximately one week before the onset of neurological symptoms). In the VA7-EGFP-treated mice, Fluc signal was completely quenched within 6 days after virus injection, remaining undetectable in all 5 mice during the 100-day follow-up (Figure 4). Neither weight loss nor neurological symptoms were observed, and we also did not find any persistent virus in the CNS of these animals by titration (data not shown).

### Replication of VA7-Rluc in Naïve and Tumor-Bearing Mice

To study the virus biodistribution we infected 11 naïve mice with 1×10^6^ PFU of VA7-Rluc. The animals were sacrificed at days 3, 6 and 9 post infection and brains, heart, liver and spleen were removed for titration and histology. Virus was detected in all analyzed organs at day 3 but exclusively in the brain at day 6. No virus was recovered from any organ beyond this time point (Table 1). Histological assessment of the organs at day 3 revealed no histopathological changes (Figure S7).

**Table 1:** Viral titers in organs from VA7-Rluc–treated nude mice.

	Day 3 (n = 4)	Day 6 (n = 4)	Day 9 (n = 3)	Day 90 (n = 5)
Brain	5.8×10^2^ (40–1.2×10^3^)	2×10^4^ (8×10^3^–3×10^4^)	ND	ND
Heart	1×10^4^ (1×10^4^–1.1×10^4^)	ND	ND	ND
Liver	6.3×10^3^ (9×10^2^–1.9×10^4^)	ND	ND	ND
Spleen	1.9×10^4^ (4.8×10^3^–4.7×10^4^)	ND	ND	ND

Viral titer (PFU/g tissue).

ND = not detected.

Data from mice sampled at day 90 are from in vivo experiment shown in Figure S6.

**Figure 5:**
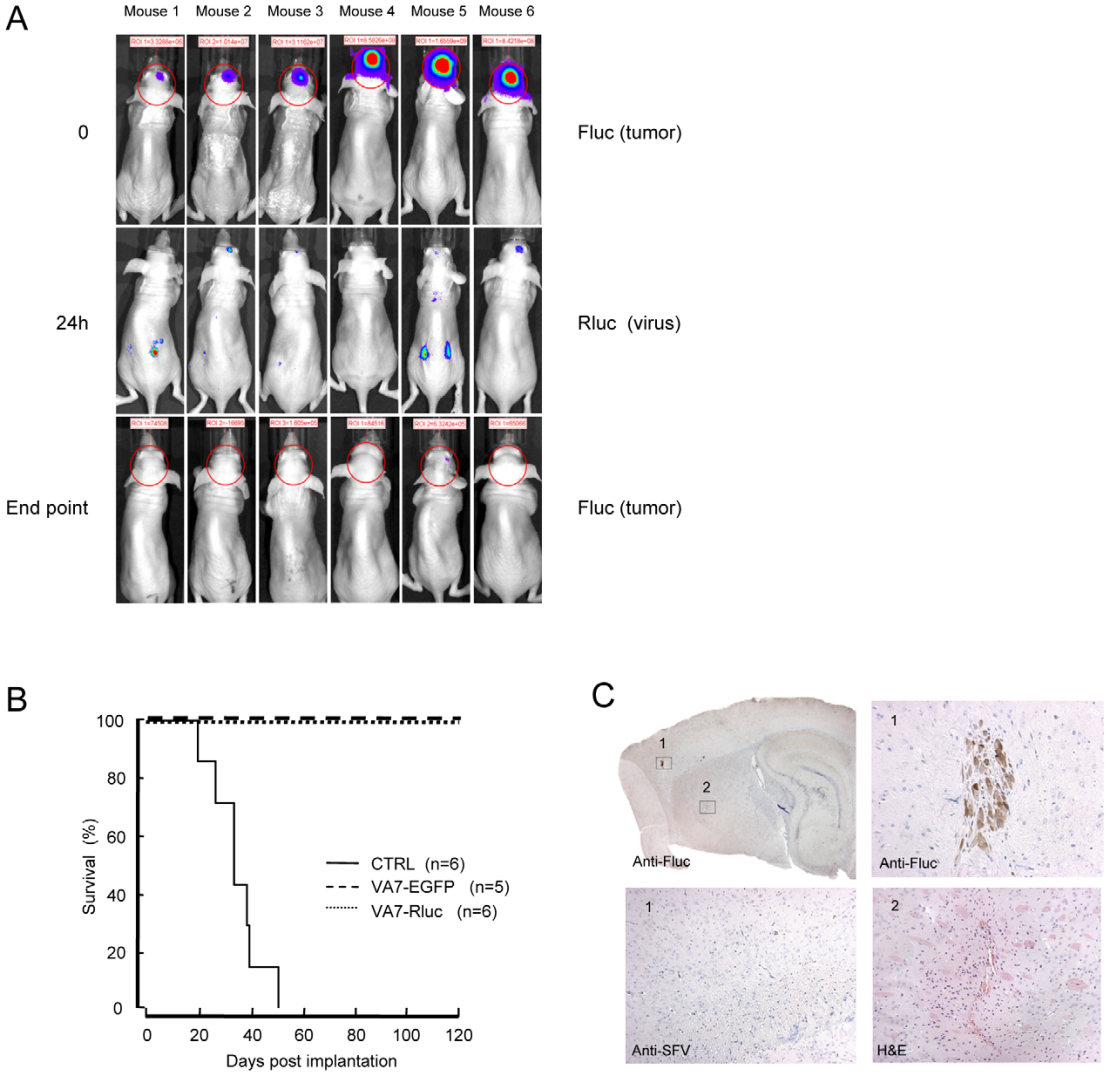
A single intravenous injection of VA7-Rluc has potent antiglioma effect in nude mice bearing orthotopic U87Fluc tumors. (A) Orthotopic U87Fluc xenografts were established in Balb/c nude mice by stereotactic injections of the tumor cells. When the firefly bioluminescence from the tumor xenografts reached appropriate levels the animals were given an intravenous injection of 1×10^6^ PFU of VA7-Rluc. After 24 hours, the animals were injected intravenously with ViviRen substrate and the light produced by the VA7Rluc vector was monitored by a cooled CCD camera. Transient virus replication (Rluc) could be detected in the brain, co-localizing with the tumor (Fluc), as well as in the periphery. Even tumors close to endpoint were fully eradicated. Virus signals can be compared with each other but not with tumor signal due to different intensity scale. (B) Long-term survival of mice in Figure 4 and Figure 5A. None of the animals treated intravenously with either VA7-EGFP or VA7-Rluc reached the end point bioluminescence value. The median survival of PBS-treated (CTRL) mice was 36 days (95% CL = 26 days to 42 days) and all differences to this group were highly significant (log-rank test, *P*<0.0001). (C) Sagittal section of the brain of mouse 5 stained against Fluc (*top left*). This mouse was given a single intravenous dose of 1×10^6^ PFU of VA7-Rluc and was the only one in the group of intravenously -treated mice that produced an intracranial Fluc IVIS signal at the final measurement of the experiment. *Top right*, 20× magnification of box 1 showing a Fluc-positive cell cluster. *Bottom left*, 10× magnification of box 1 showing anti-SFV immunostaining of parallel brain section from the same mouse. No SFV immunoreactivity could be detected anywhere in the brain of this or any other i.v. treated mouse. *Bottom right*, 10× magnification of the region in box 2 from a parallel section stained with H&E. The increased cellular density surrounding the blood vessel in the picture is indicative of regenerative activity. This region was possibly the site of the original tumor before destruction by the VA7-Rluc virus.

To relate the viral titers to ectopic expression of *Renilla* luciferase we injected 6 mice bearing intracranial tumors with VA7-Rluc. We used ViviRen substrate, shown to give bioluminescence signals up to three times higher than coelenterazine, the natural substrate of Rluc [33]. Between 16 and 40 hours post injection, five animals exhibited Rluc which co-localized with tumor-derived Fluc (for complete time course see Figure S4). In four animals transient Rluc expression could be observed in peripheral organs (Figure 5A). Tumor signal was undetectable in all mice by 4 days post injection, but reappeared in one mouse (mouse 1) at day 24, only to regress again and remain absent at the final measurement 101 days post injection (Figure S4). All the animals treated intravenously with either VA7-EGFP or VA7-Rluc virus were alive at 120 days post implantation (Figure 5B), and all except one (mouse 5) scored negative for tumor signal. Post mortem histology of the brain of this Fluc-positive animal revealed one larger cluster of firefly luciferase expressing tumor cells (Figure 5C). An ongoing wound healing process was also apparent, although at a relatively long distance from the residual tumors (Figure 5C). No Fluc positive cells were found in the brains of the other 5 animals and in addition to mouse 5, signs of tissue trauma were evident also in the brain of mouse 6 (Figure S5). In both cases, these regions were most likely the original tumor sites. We also examined the brains of all tumor-bearing mice which had received VA7-Rluc for the presence of SFV antigen, but no persistent virus could be detected (result shown for mouse 5 in Figure 5C).

**Figure 6:**
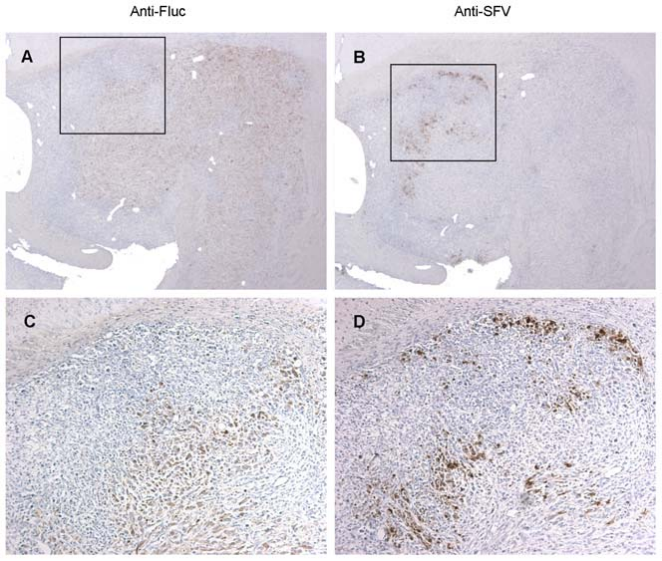
Immunohistochemical analysis of VA7 replication in intracranial U87Fluc xenografts. U87Fluc cells were implanted in the striatum Balb/c nude mice and tumor growth was monitored by magnetic resonance imaging. When tumors were visible mice were injected with 1×10^6^ pfu of VA7-EGFP and sacrificed three days later. Brains were removed and paraffin sections prepared. (A–D) Representative images of U87Fluc xenograft immunostained against Fluc (A,C) and SFV antigen (B,D). (A,B) magnification, ×27; (B,D) 3.7× magnification of boxed areas in (A,B).

We repeated the survival experiment with similar results. Only one out of 6 U87Fluc implanted and VA7Rluc –treated mice showed tumor regrowth after initial tumor regression. All other 5 animals survived 90 days after virus injection and histological assessment revealed no pathological changes in the organs studied (Fig. S7). Also, we were unable to detect virus in the treated mice at day 90 (Table 1). In contrast, immunohistochemical analysis of three tumor-bearing animals sampled at day 3 post virus injection revealed disseminated virus antigen in the intracranial tumors thus confirming productive infection of glioma xenografts (Fig. 6).

## Discussion

In the present paper we describe the oncolytic potential of the SFV vector VA7 in a nude mouse model of human glioma. The initial results that small subcutaneous tumors were completely eradicated by a single injection of VA7-EGFP were promising, but regrowth after initial regression of larger tumors was suggestive of emerging resistance, as seen in a human melanoma model [32]. However, as the isolated explant cultures could be killed by VA7-EGFP, mechanisms other than resistance to VA7 are responsible for the failure to achieve complete eradication of large tumors.

The observation that larger tumors do not respond as well as smaller tumors to oncolytic virus is not new [34]. For example, subcutaneous rhabdomyosarcoma [35], human lymphoma xenografts [36] and orthotopic gliomas [37] showed reduced response as tumor size increased. Apart from tumor-related barriers, such as connective tissue and increased interstitial pressure, the innate immune system is known to restrict virus spread and to cause premature virus clearance [34], [38]. While SFV establishes a viremia that lasts up to 4 days, even nude mice clear the virus with kinetics similar to immunocompetent animals, mediated by serum IgM [39]. Because attenuated SFV is cleared at a slower rate from the brain [29] it may be particularly well suited to target intracranial tumors.

A major reason for glioma recurrence is the invasion of the healthy brain parenchyma [40]. Although the U87 nude mouse model is not generally considered invasive, sporadic tumor cells have been detected outside the main tumor mass and the model does recapitulate some of the other characteristics of human malignant glioma [12], [41]. Also, the gene expression of U87 and U251 cells change towards a common profile when they are implanted orthotopically rather than subcutaneously [42], and only in orthotopically implanted U87 xenografts can purported CD133-positive brain tumor stem cells be detected [43]. It was therefore encouraging to observe that a single intravenous injection of the VA7 virus was sufficient to fully cure the vast majority of the animals harboring orthotopic xenografts. Only one animal out of 17 intravenously-treated mice relapsed and only in one mouse residual tumor cells could be detected at the study end point. In comparison, intratumoral administration of reovirus [7], myxomavirus [14], intergeneric poliovirus [15] or chimeric HSV-1 [44] has produced long term, tumor-free survivors in the range of 75 to 100%, but where studied, peripheral administration has produced none. Mutant VSV was recently shown to retard the growth of U87 orthotopic tumor xenografts after intravenous administration but all the animals eventually died of recurrent tumors [12]. This was not likely due to poor virus penetration into the tumors, as in another study systemic administration of VSV effectively induced apoptosis in virtually all cells of orthotopic glioma xenografts [45].

It remains unclear whether the remaining Fluc-positive cells in the sole mouse displaying Fluc-signal at study end point (Figure S4, mouse 5) could have formed a viable tumor, as another tumor-bearing animal treated with VA7-Rluc and apparently tumor-free (Figure S4, mouse 1) presented later with recurrent Fluc signal that, however, eventually cleared and no remaining tumor cells were seen in the brain at the end of the study. Although we do not exclude the possibility that persisting virus in the brain re-infected the residual cells in this mouse, in contrast to previous reports using SFV A7(74) [29], [39]. We could not find any evidence of VA7 virus persistence in the brains of the nude mice. This discrepancy might be explained by genetic differences between the cloned VA7 virus and the parental virus [28], [31].

A potential limitation of using systemic delivery of oncolytic viruses is replication outside the target tissue. In the present study, Renilla bioluminescence indicative of VA7 replication could be detected outside tumor tissue indicating that VA7 does not show strict tumor specificity which is not unexpected. This appears to be the case even for SFV replicons [46]. We could not detect any *Renilla* bioluminescence in naïve mice (data not shown) which suggests that the sensitivity of Rluc imaging is not high enough to detect such low levels of virus as present in the organs of naïve mice. Plaque assay, however, revealed transient presence of virus in all analyzed organs but none of the treated animals displayed any neurological symptoms of infection and no histopathological changes were observed (Figure S7) showing that VA7 virus is a safe vector in adult hosts. This is corroborated by numerous studies both in animals and CNS model systems using parental SFV A7(74) [26], [28], [29], [39].

In conclusion, we have shown that replication-competent vector VA7 based on attenuated SFV effectively infects and kills human glioma tumors in nude mice while leaving healthy brain tissue unharmed. We want to stress, however, that because of the extreme susceptibility to VA7 of U87 cells, our experiments may give an overestimation of the oncolytic efficacy of this virus and should therefore be considered the best case scenario. Thus, this work is the prelude to studies addressing the oncolytic capacity of attenuated SFV in the presence of an intact immune system. If the results of this study can be validated in immunocompetent models, the possibility to administer the virus systemically and its natural capacity to pass the blood-brain-barrier are attractive features that may prove beneficial in clinical settings.

## Materials and Methods

### Ethics Statement

All animal work has been conducted according to relevant national and international guidelines. Approval has been obtained from the Finnish National Review Board for Animal Experimentation.

### Cell Lines

U87MG and A172 human glioma cell lines were obtained from the American Type Culture Collection (ATCC). U251 human glioma cells were obtained from Dr. J. Wahlfors (A.I. Virtanen Institute, Kuopio, Finland). The cells were cultured in Dulbecco's modified Eagle's medium containing 10% fetal calf serum at 37°C in 5% CO_2_. U87Fluc cells were established by lentiviral transduction using WPTFluc, constructed by replacing the GFP gene in WPTGFP (from Dr. D. Trono) with the luciferase gene from pMIR-REPORT™ plasmid (Ambion Inc., TX, USA).

### Construction and Production of VA7 Vectors

Construction and production of VA7-EGFP has been described [31]. VA7-Rluc was constructed by cutting the gene encoding *Renilla* luciferase from pRL-Null vector (Promega, Madison, WI, USA) with *Xb*aI and *Nhe*I and blunt-cloning it into the pSTBlue-1 vector (Novagen, Madison, WI, USA), from which it was cut out using *BamH*I and *Avr*II and then inserted into the VA7 vector cut with the same enzymes.

### Cell Viability Assay

Glioma cells were plated at 3×10^3^ cells per well on a 96-well plate. VA7-EGFP at MOI 10^−5^–10^1^ was added 16 hours later in a volume of 10 µl and the cells were incubated for 96 hours. Calcein AM was added to a final concentration of 1 µM and the cells were incubated for 12 minutes at 37°C, after which the wells were rinsed once with PBS and finally, 50 µl of PBS was pipetted to each well and fluorescence was measured with Victor 1420 Multilabel Counter (Perkin-Elmer Wallac, Turku, Finland).

### Immunodetection of VA7-EGFP Structural Proteins

Glioma cells were seeded at 2×10^5^ cells/well on a 6-well plate. Twenty hours later VA7-EGFP was added at an MOI of 1. After 18 hours the medium was removed and the samples were processed for Western analysis as described in [28].

### Virus Titration

MBA-13 cells were seeded on 6-well plates at 3×10^5^/well. Tissue homogenates were added 24 hours later and incubation was continued for one hour. The medium was then replaced with a medium containing 0.5% FCS and 0.4% SeaPlaque agarose. After 48 hours the agarose was removed and the cell monolayers fixed and stained with crystal violet solution (0.2% crystal violet w/v, 5% formaldehyde and 1% ethanol).

### Animals

Seven-to-eight-week-old female Balb/cOlaHsd Foxn1−/− mice were purchased from Harlan Laboratories (Allerod, Denmark) and housed in filter cages in groups of 4 mice per cage. Animal experiments were conducted in Turku and Kuopio, Finland, under experimental protocols approved by the Finnish National Review Board for Animal Experimentation.

### Evaluation of VA7-EGFP Anti-Glioma Activity in Subcutaneous Tumor Model

To induce subcutaneous tumors, 5×10^6^ U87Fluc cells in 100 µl of PBS were injected into the right flanks of nude Balb/c mice. Tumor length and width were measured every third day with a caliper and the tumor volumes were calculated from the equation (length×width^2^)/2. When the tumors reached ∼100 mm^3^, four mice received a single intravenous injection of PBS (control). A group of 5 mice received a single intravenous injection of 1×10^6^ PFU of VA7-EGFP. The rest of the animals were left untreated until the average tumor size was 450 mm^3^, at which point a group of 6 mice received a single intravenous injection of 1×10^6^ PFU VA7-EGFP while another group of 6 mice received three intravenous injections of VA7-EGFP, each 1×10^6^ PFU, at 7-day intervals. Additional 7 mice with tumor sizes averaging 450 mm^3^ received either a single or three injections of VA7-EGFP and were used for histological studies. Animals were killed with CO_2_ when the tumor size reached 800 mm^3^.

### Evaluation of VA7-EGFP Anti-Glioma Activity in Intracranial Tumor Model

U87Fluc cells were cultured as described above. Cells were detached with trypsin/EDTA, spun down, washed with PBS and suspended in PBS at 2×10^5^ cells/3 µl. Mice were anesthetized with an intraperitoneal injection of ketamine (75 mg/kg) plus medetomidine (1 mg/kg). The animals were placed on a stereotactic apparatus (Stoelting, Wood Dale, IL, USA), the head was cleaned with 70% isopropanol solution and an incision was made along the midline. The skin was withdrawn to the sides and a burr hole with a diameter of 0.5 mm was drilled 0.5 mm anterior and 1.5 mm lateral to bregma. A 10 µl Hamilton syringe with a 30 gauge needle was mounted on the stereotactic frame and lowered to a depth of 3 mm at 1 mm/min. Two hundred thousand cells in 3 µl PBS were injected at 1 µl/min. After the injection was completed, the needle was kept in place for additional two minutes and then slowly withdrawn. The skin folds were closed with polyamide surgical thread and the animals were aroused with an i.p. injection of atipamezole (Antisedan, 1 mg/kg). Three weeks later the mice were imaged for intracranial Fluc activity. Mice with intracranial bioluminescence were randomly divided into three groups. A group of 6 mice was injected with PBS while another group of 5 mice received a single intravenous dose of 1×10^6^ pfu of VA7-EGFP. Using the PBS group, an IVIS end point for sacrifice of the mice (1×10^9^ photons/sec/cm^2^/steradian) was set to roughly one week before the appearance of clinical signs due to tumor burden.

### VA7-Rluc Replication in Tumor-Bearing Nude Mice

U87FLuc tumor cells were implanted intracranially in 6 nude mice as described above and imaged for the presence of intracranial firefly bioluminescence 16 days later. Three bioluminescence positive mice were injected intravenously with 1×10^6^ PFU of VA7-RLuc. The mice were imaged for *Renilla* bioluminescence at 16, 24, 40 and 96 hours post injection. After the last time point when Rluc signal had abated, the mice were imaged for Fluc activity and followed regularly until the termination of the study. Tumor signal in the remaining three tumor-bearing mice was monitored regularly until close to the IVIS endpoint upon which the mice received a single intravenous injection of 1×10^6^ pfu of VA7-Rluc and were imaged as above. The experiment was repeated with 6 tumor-bearing mice which received a single injection of VA7-Rluc but this time only firefly bioluminescence was monitored.

### *In vivo* Imaging

For detection of Fluc activity, mice were injected intraperitoneally with 150 µl of beetle luciferin (Promega, Madison WI, USA) at 2 µg/µl and anesthetized with a continuous 2.5% isoflurane flow. Ten minutes later, the animals were moved into IVIS 50 (Xenogen) apparatus chamber with ongoing anesthesia and 30 second images were acquired. For detection of VA7-Rluc, mice were injected intravenously with 100 µl of ViviRen substrate (Promega) at 0.2 µg/µl in 0.1% bovine serum albumin-PBS under continuous isoflurane anesthesia. Mice were imaged two minutes after substrate injection using data acquisition time of 3 minutes.

### Histology

Mice were CO_2_ -euthanized and perfused intracardially with PBS. Organs were removed, fixed in paraformaldehyde, embedded in paraffin, sectioned and stained with hematoxylin and eosin, rabbit anti-SFV antibody (1∶3000) and polyclonal anti-luciferase antibody (1∶250, MBL Medical & Biological Laboratories Ltd, Nagoya, Japan) as described [28].

### Statistical Analyses

Survival curves were generated by the Kaplan – Meier method and the log-rank test was used to compare groups. All reported *P* values are two-sided and are considered to be statistically significant at *P*<0.05.

## Supporting Information

Figure S1 Characterization of Fluc expressing U87 glioma cells. A) U87 cells were transduced with the lentiviral vector WPTFluc at MOI 1 to 50 and the luciferase activities of the transduced cells were determined. B) U87 cells expressing Fluc were seeded on a 96-well plate and proliferation of the cells was measured during a 5-day period using calcein AM probe as an indicator of viable cells. Results are shown as the means and 95% confidence intervals. C) Oncolytic activity of VA7-EGFP on the parental U87 cells and cells which had been transduced with WPTFluc at MOI 25 (U87Fluc25) was investigated. VA7-EGFP was added to cells at MOI 10−3 to 1. Cell viability was measured 96 hours later using the calcein AM probe. The results are shown as the means and 95% confidence intervals. D) Flow cytometry analysis of U87 and U87Fluc cells detected with anti-luciferase antibody and Alex-488 -coupled secondary antibody.
(0.29 MB TIF)

Figure S2 Effect of intravenously administered VA7-EGFP on the weight of nude mice. Balb/c nude mice received a single intravenous injection of either PBS (n = 3) or 1×1E6 PFU of VA7-EGFP (n = 3). The animals were weighed every 6th day during a 48-day period. During this time period no neurological symptoms or other adverse effects in the virus-treated group was observed. The results are shown as the means and 95% confidence intervals.
(0.05 MB TIF)

Figure S3 Characterization of tumor cell explants from subcutaneous U87Fluc tumors. A) Cell lines established from subcutaneous U87Fluc tumors that had progressed after initial response were grown on cover slips and stained with anti-luciferase antibody followed by detection with Alexa 488-coupled secondary antibody. Left, fluorescence; right, phase contrast photomicrographs. B) Eight cell lines derived from 7 different subcutaneous tumors, which had regrown after VA7-EGFP treatment, were exposed to VA7-EGFP at increasing MOI. Also two cell lines derived from tumors of two PBS-treated mice as well as the parental cell line U87Fluc were exposed to the virus. Cell viability was measured 96h later using calcein AM probe. Sensitivities for VA7-EGFP-induced cell killing of the cells derived from VA7-EGFP-treated tumors fell between those of U87Fluc cells and cells derived from tumor of a PBS-treated mouse. Each data point is the mean of 6 determinations and the error bars are not shown for the sake of clarity.
(2.49 MB TIF)

Figure S4 VA7-Rluc replication in U87Fluc tumor-bearing mice. Orthotopic U87Fluc xenografts were established in nude mice by stereotactic injections of the tumor cells. When the firefly bioluminescence from the tumor xenografts reached appropriate levels the animals were given an intravenous injection of 1×1E6 PFU of VA7 expressing Renilla luciferase. After either 16 or 24 hours the animals were injected intravenously with Viviren substrate and the light produced by the VA7Rluc vector was monitored by a cooled CCD camera. Transient virus replication (Rluc) could be detected in the brain, co-localizing with the tumor (Fluc), as well as in the periphery. Virus signals can be compared with each other but not with tumor signal due to different intensity scale. Tumor signal was extinguished within 4 days, irrespective of the size of the tumor at the start of the treatment. In all but one mouse (nr. 5), tumor signal remained below detection limit at the end of the study. In another mouse (nr. 1), tumor regrowth followed by remission could be detected after day 4 post injection.
(4.04 MB TIF)

Figure S5 Hematoxylin and eosin staining of brain tissue of VA7-Rluc-treated mice 120 days after U87Fluc tumor implantation. After the final *in vivo* imaging the mice shown in figure 5 were intracardially perfused with PBS, the brains were removed and fixed in formalin and embedded in paraffin. Sagittal sections were prepared and stained with hematoxylin and eosin. For mouse 5, which was the only Fluc positive animal at the final measurement, a section taken 100 µm mediolateral from the one shown in Figure 5 is presented here. Also in this section signs of tissue defects are evident (boxed area) although no residual tumor cells can be distinguished. A tissue trauma is also evident in the brain of mouse 6 (boxed area). This site is most likely the original tumor site but no tumor cells can be detected in this section and nor were Fluc cells detected in other sections from this animal either. For mice 5 and 6 a 13× magnification of the trauma sites are shown on the right. Bar = 1.0 mm.
(5.61 MB TIF)

Figure S6 Effect of a single injection of VA7-Rluc on the growth of intracranial U87Fluc xenografts. U87Fluc cells were implanted stereotactically in Balb/c nude mice and the tumor growth was monitored by IVIS Imaging system. Eighteen days later the animals were given a single injection of 1×1E6 pfu of VA7-Rluc or PBS (controls). Of the virus-treated mice only one developed tumor after initial tumor regression (mouse 1). Rest of the animals stayed tumor-free for the rest of the follow-up period. Fluc bioluminescence in PBS-treated mice reached end point level in two weeks.
(3.61 MB TIF)

Figure S7 Hematoxylin and eosin staining of organs from VA7-treated mice. Left panel, Balb/c nude mice were injected with 1×1E6 PFU of VA7-Rluc and the animals were sacrificed at days 3, 6 and 9 after infection. Brain, heart, liver and spleen were removed for titration and histological analysis. Representative images of HE-stained organs from a mouse sampled at day 3 post infection. Right panel, organs from the VA7-Rluc-treated mice from the *in vivo* experiment shown in Figure S6 were removed at the termination of the study and cut in half. One half was frozen in liquid nitrogen for plaque assay and the other half was fixed in paraformaldehyde and processed for hematoxylin and eosin staining. Representative HE images from mouse 3 are shown. Original magnifications: brain×100, heart, spleen and liver×200.
(13.82 MB TIF)
