# Supplementary figures and images for: Intravenously Administered Alphavirus Vector VA7 Eradicates Orthotopic Human Glioma Xenografts in Nude Mice
Source: PLoS One. 2010 Jan 6;5(1):e8603. doi: 10.1371/journal.pone.0008603 (PMC2799335; doi:10.1371/journal.pone.0008603)

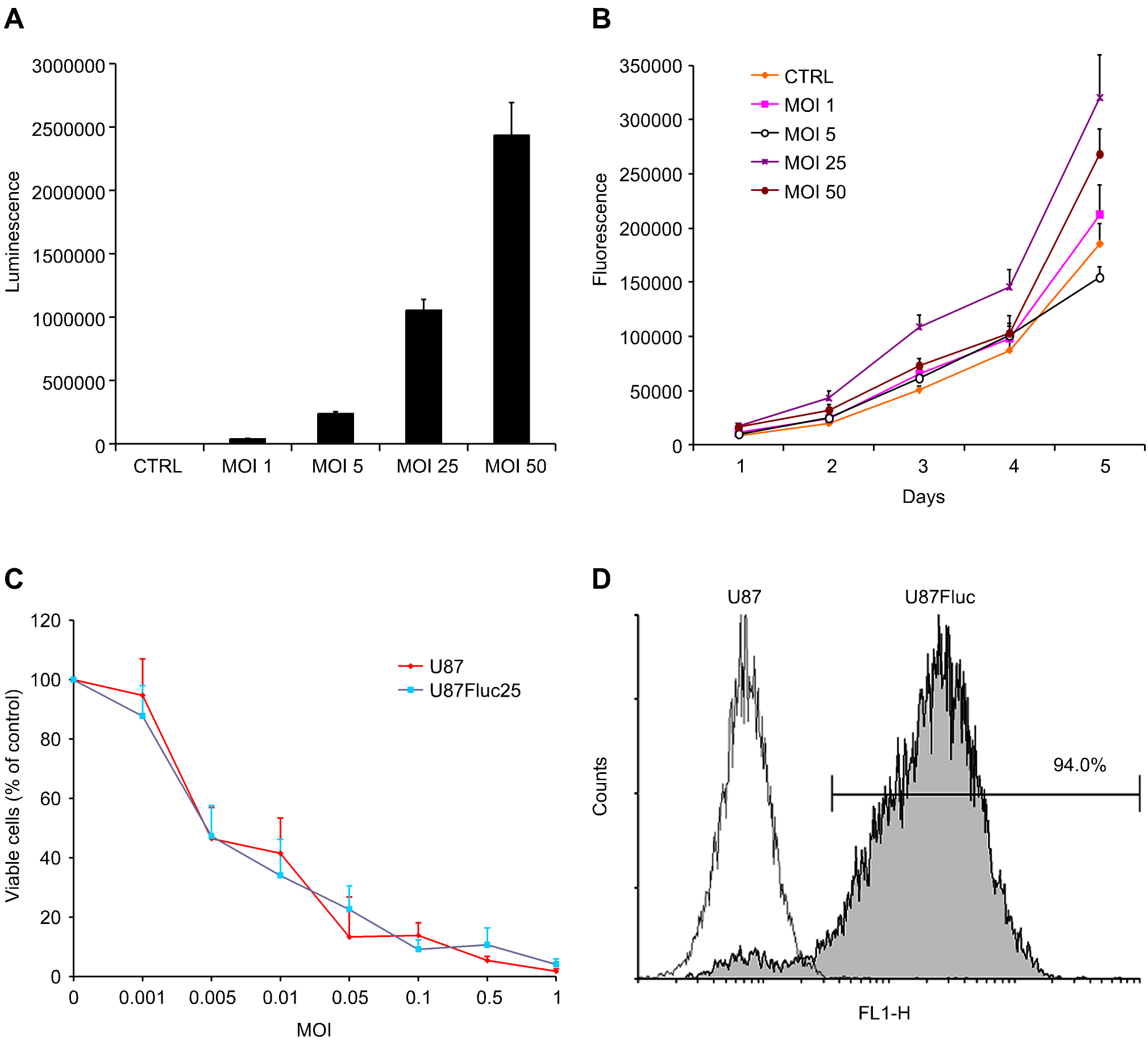

Supplement: Figure S1 — Characterization of Fluc expressing U87 glioma cells. A) U87 cells were transduced with the lentiviral vector WPTFluc at MOI 1 to 50 and the luciferase activities of the transduced cells were determined. B) U87 cells expressing Fluc were seeded on a 96-well plate and proliferation of the cells was measured during a 5-day period using calcein AM probe as an indicator of viable cells. Results are shown as the means and 95% confidence intervals. C) Oncolytic activity of VA7-EGFP on the parental U87 cells and cells which had been transduced with WPTFluc at MOI 25 (U87Fluc25) was investigated. VA7-EGFP was added to cells at MOI 10−3 to 1. Cell viability was measured 96 hours later using the calcein AM probe. The results are shown as the means and 95% confidence intervals. D) Flow cytometry analysis of U87 and U87Fluc cells detected with anti-luciferase antibody and Alex-488 -coupled secondary antibody. (0.29 MB TIF) [file pone.0008603.s001.tif]

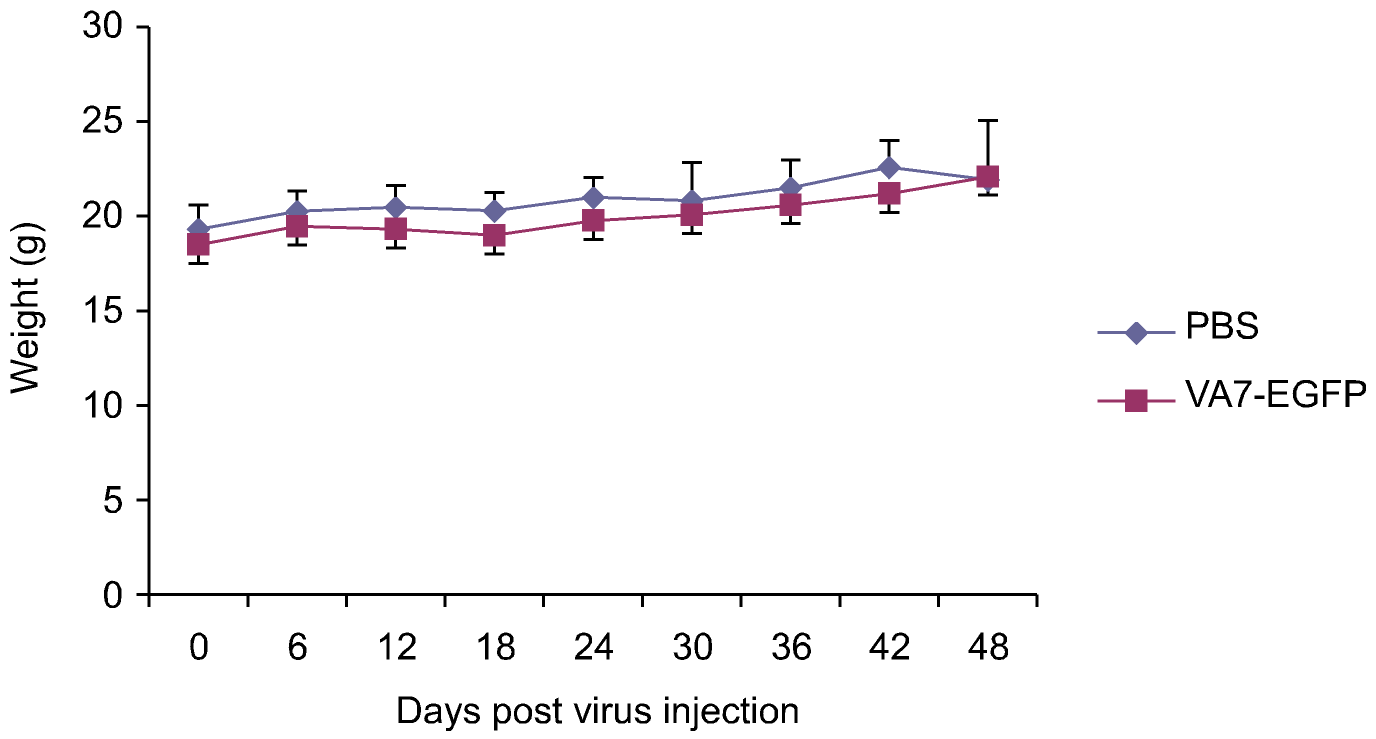

Supplement: Figure S2 — Effect of intravenously administered VA7-EGFP on the weight of nude mice. Balb/c nude mice received a single intravenous injection of either PBS (n = 3) or 1×1E6 PFU of VA7-EGFP (n = 3). The animals were weighed every 6th day during a 48-day period. During this time period no neurological symptoms or other adverse effects in the virus-treated group was observed. The results are shown as the means and 95% confidence intervals. (0.05 MB TIF) [file pone.0008603.s002.tif]

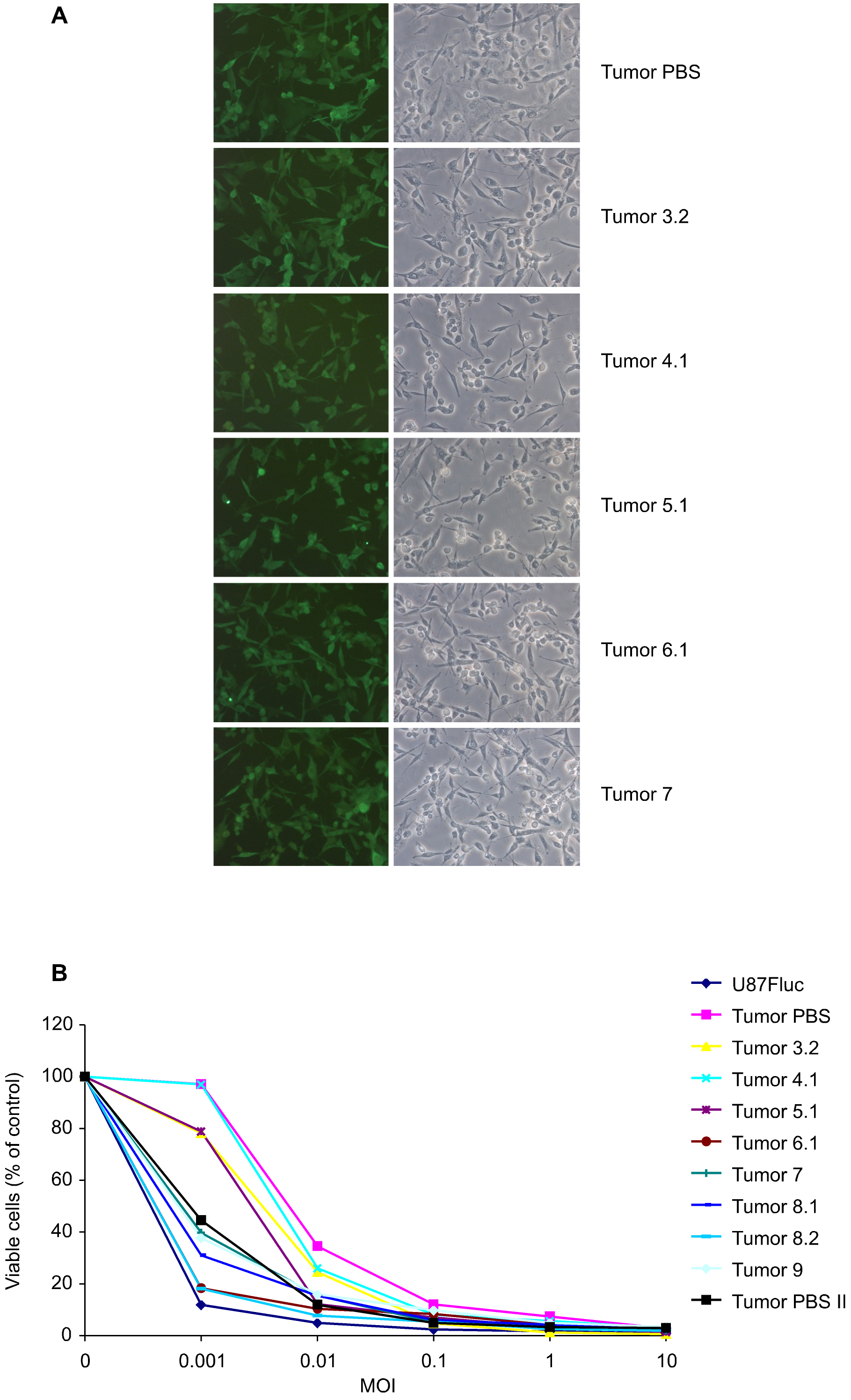

Supplement: Figure S3 — Characterization of tumor cell explants from subcutaneous U87Fluc tumors. A) Cell lines established from subcutaneous U87Fluc tumors that had progressed after initial response were grown on cover slips and stained with anti-luciferase antibody followed by detection with Alexa 488-coupled secondary antibody. Left, fluorescence; right, phase contrast photomicrographs. B) Eight cell lines derived from 7 different subcutaneous tumors, which had regrown after VA7-EGFP treatment, were exposed to VA7-EGFP at increasing MOI. Also two cell lines derived from tumors of two PBS-treated mice as well as the parental cell line U87Fluc were exposed to the virus. Cell viability was measured 96h later using calcein AM probe. Sensitivities for VA7-EGFP-induced cell killing of the cells derived from VA7-EGFP-treated tumors fell between those of U87Fluc cells and cells derived from tumor of a PBS-treated mouse. Each data point is the mean of 6 determinations and the error bars are not shown for the sake of clarity. (2.49 MB TIF) [file pone.0008603.s003.tif]

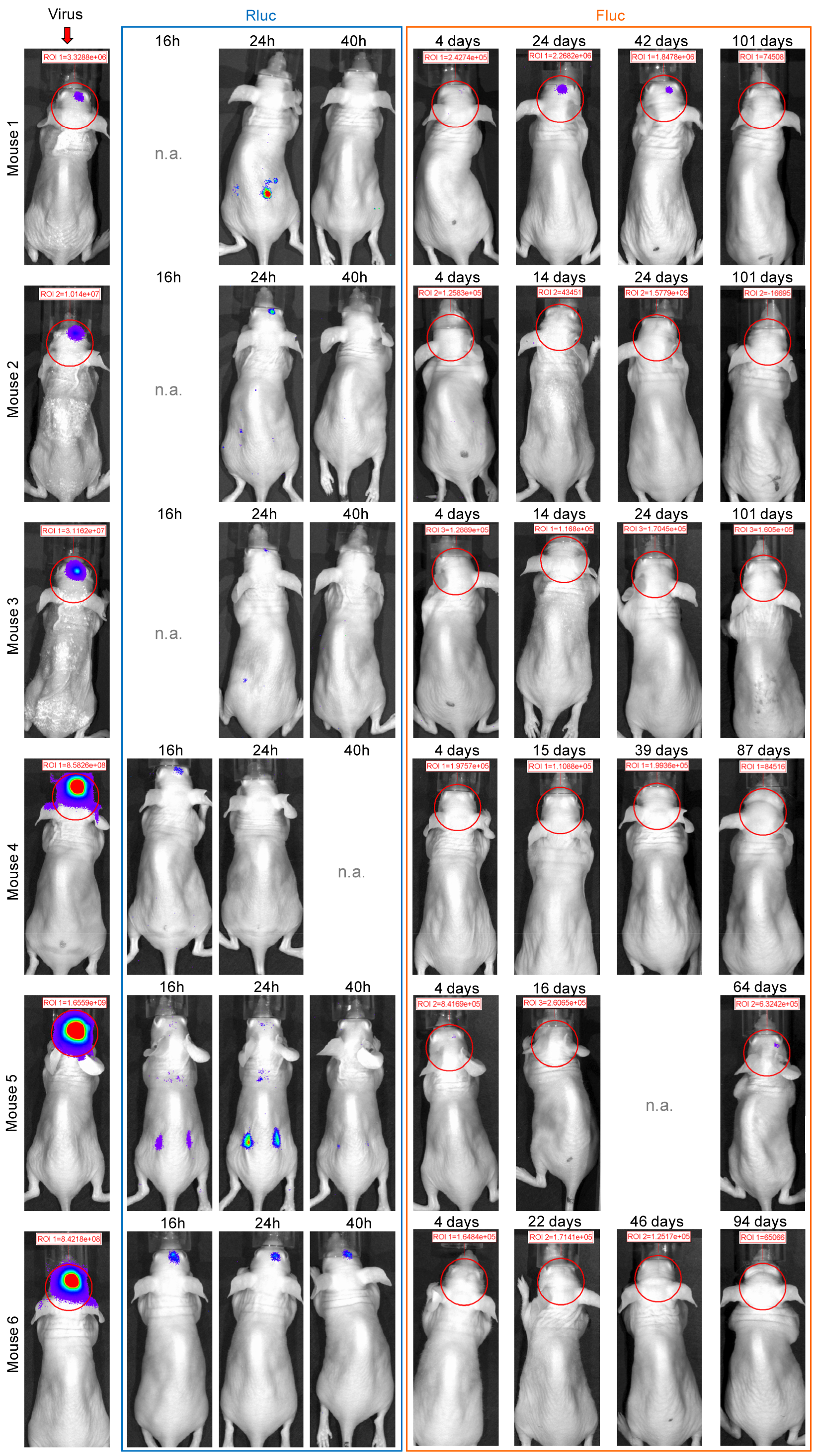

Supplement: Figure S4 — VA7-Rluc replication in U87Fluc tumor-bearing mice. Orthotopic U87Fluc xenografts were established in nude mice by stereotactic injections of the tumor cells. When the firefly bioluminescence from the tumor xenografts reached appropriate levels the animals were given an intravenous injection of 1×1E6 PFU of VA7 expressing Renilla luciferase. After either 16 or 24 hours the animals were injected intravenously with Viviren substrate and the light produced by the VA7Rluc vector was monitored by a cooled CCD camera. Transient virus replication (Rluc) could be detected in the brain, co-localizing with the tumor (Fluc), as well as in the periphery. Virus signals can be compared with each other but not with tumor signal due to different intensity scale. Tumor signal was extinguished within 4 days, irrespective of the size of the tumor at the start of the treatment. In all but one mouse (nr. 5), tumor signal remained below detection limit at the end of the study. In another mouse (nr. 1), tumor regrowth followed by remission could be detected after day 4 post injection. (4.04 MB TIF) [file pone.0008603.s004.tif]

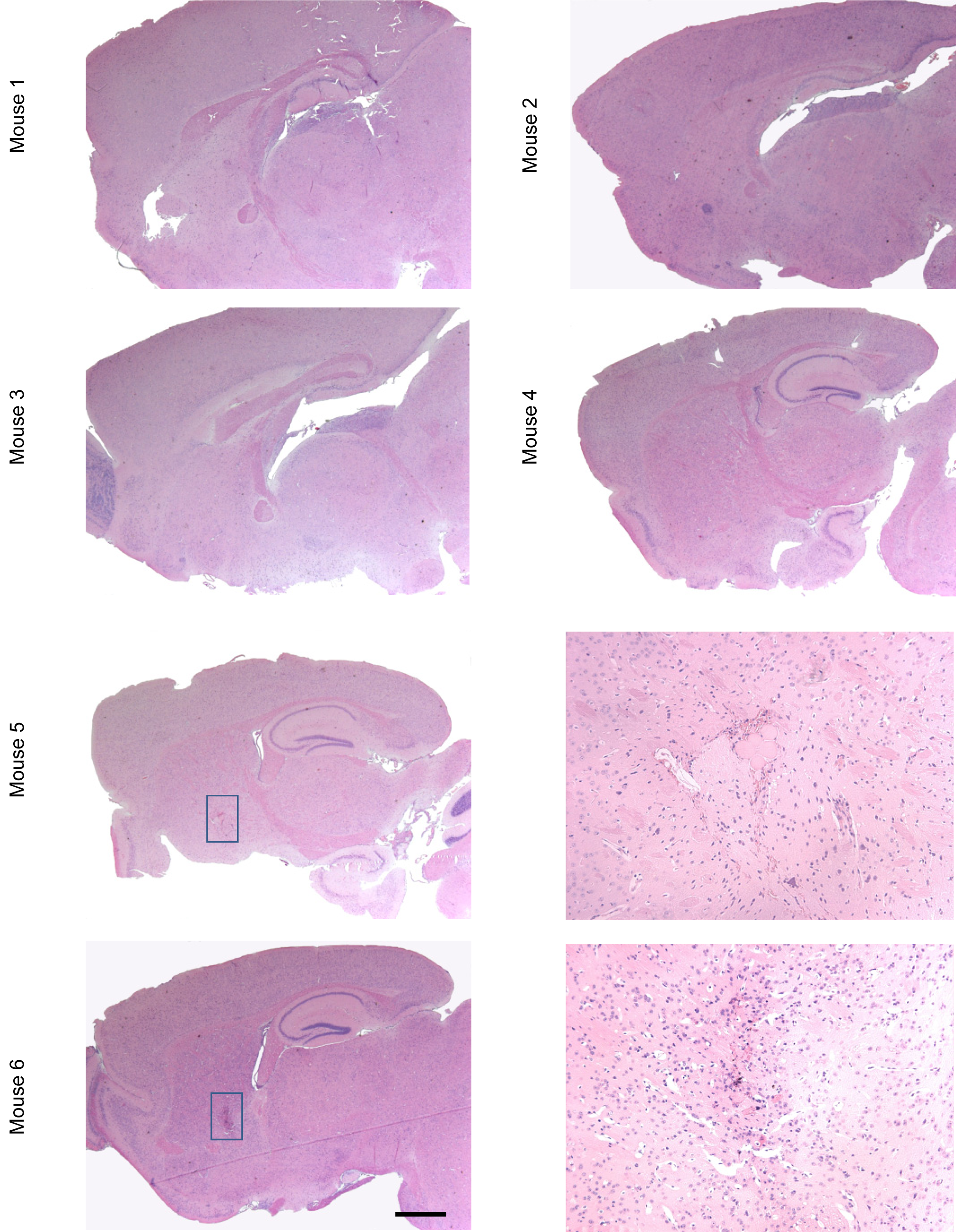

Supplement: Figure S5 — Hematoxylin and eosin staining of brain tissue of VA7-Rluc-treated mice 120 days after U87Fluc tumor implantation. After the final in vivo imaging the mice shown in figure 5 were intracardially perfused with PBS, the brains were removed and fixed in formalin and embedded in paraffin. Sagittal sections were prepared and stained with hematoxylin and eosin. For mouse 5, which was the only Fluc positive animal at the final measurement, a section taken 100 µm mediolateral from the one shown in Figure 5 is presented here. Also in this section signs of tissue defects are evident (boxed area) although no residual tumor cells can be distinguished. A tissue trauma is also evident in the brain of mouse 6 (boxed area). This site is most likely the original tumor site but no tumor cells can be detected in this section and nor were Fluc cells detected in other sections from this animal either. For mice 5 and 6 a 13× magnification of the trauma sites are shown on the right. Bar = 1.0 mm. (5.61 MB TIF) [file pone.0008603.s005.tif]

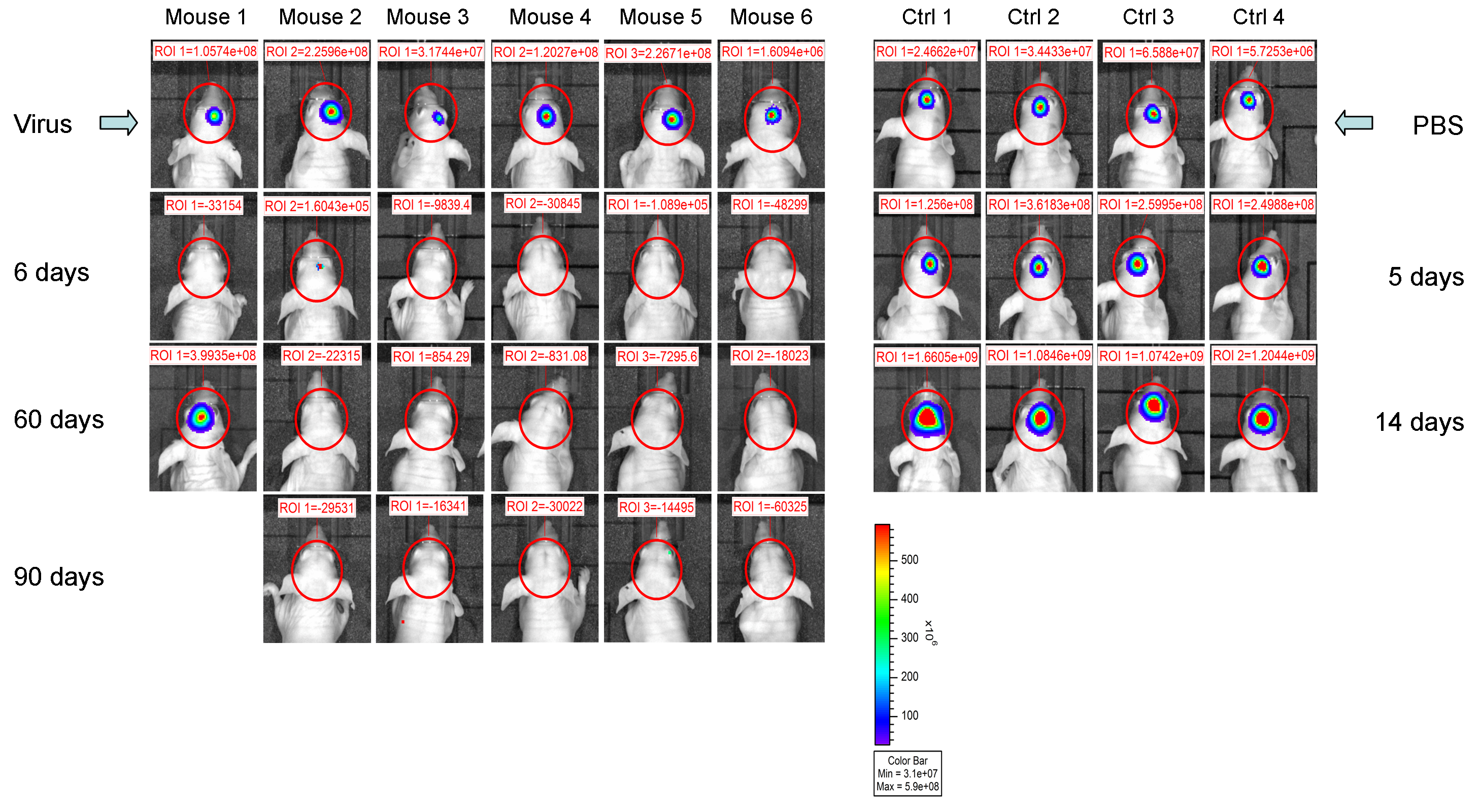

Supplement: Figure S6 — Effect of a single injection of VA7-Rluc on the growth of intracranial U87Fluc xenografts. U87Fluc cells were implanted stereotactically in Balb/c nude mice and the tumor growth was monitored by IVIS Imaging system. Eighteen days later the animals were given a single injection of 1×1E6 pfu of VA7-Rluc or PBS (controls). Of the virus-treated mice only one developed tumor after initial tumor regression (mouse 1). Rest of the animals stayed tumor-free for the rest of the follow-up period. Fluc bioluminescence in PBS-treated mice reached end point level in two weeks. (3.61 MB TIF) [file pone.0008603.s006.tif]

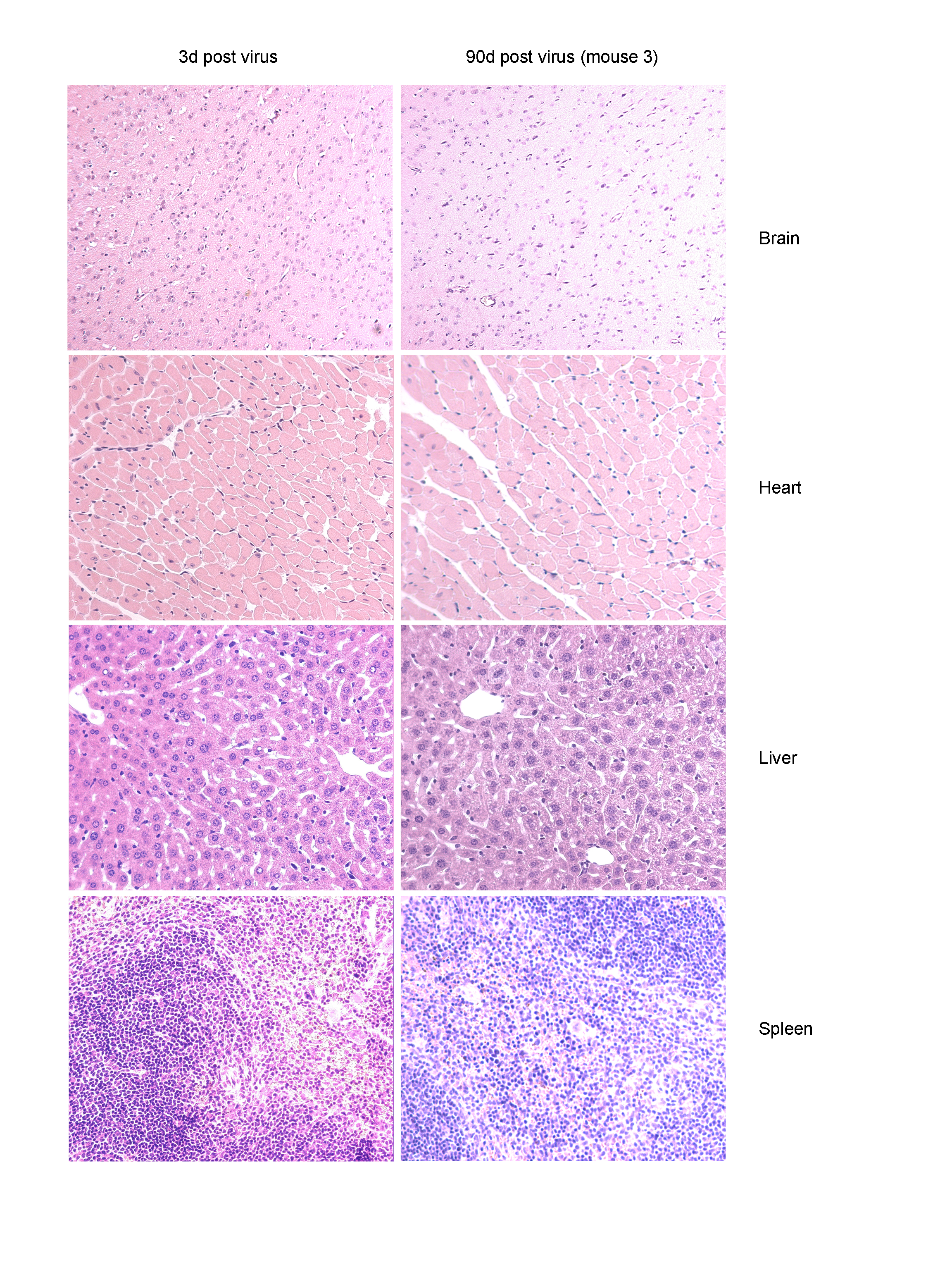

Supplement: Figure S7 — Hematoxylin and eosin staining of organs from VA7-treated mice. Left panel, Balb/c nude mice were injected with 1×1E6 PFU of VA7-Rluc and the animals were sacrificed at days 3, 6 and 9 after infection. Brain, heart, liver and spleen were removed for titration and histological analysis. Representative images of HE-stained organs from a mouse sampled at day 3 post infection. Right panel, organs from the VA7-Rluc-treated mice from the in vivo experiment shown in Figure S6 were removed at the termination of the study and cut in half. One half was frozen in liquid nitrogen for plaque assay and the other half was fixed in paraformaldehyde and processed for hematoxylin and eosin staining. Representative HE images from mouse 3 are shown. Original magnifications: brain×100, heart, spleen and liver×200. (13.82 MB TIF) [file pone.0008603.s007.tif]
